# High-throughput *Agrobacterium*-mediated barley transformation

**DOI:** 10.1186/1746-4811-4-22

**Published:** 2008-09-26

**Authors:** Joanne G Bartlett, Sílvia C Alves, Mark Smedley, John W Snape, Wendy A Harwood

**Affiliations:** 1 Department of Crop Genetics, John Innes Centre, Norwich Research Park, Colney, Norwich, NR4 7UH, UK; 2 Department of Cell and Developmental Biology, John Innes Centre, Norwich Research Park, Colney, Norwich, NR4 7UH, UK

## Abstract

**Background:**

Plant transformation is an invaluable tool for basic plant research, as well as a useful technique for the direct improvement of commercial crops. Barley (*Hordeum vulgare*) is the fourth most abundant cereal crop in the world. It also provides a useful model for the study of wheat, which has a larger and more complex genome. Most existing barley transformation methodologies are either complex or have low (<10%) transformation efficiencies.

**Results:**

A robust, simple and reproducible barley transformation protocol has been developed that yields average transformation efficiencies of 25%. This protocol is based on the infection of immature barley embryos with *Agrobacterium *strain AGL1, carrying vectors from the pBract series that contain the *hpt *gene (conferring hygromycin resistance) as a selectable marker. Results of large scale experiments utilising the *luc *(firefly luciferase) gene as a reporter are described. The method presented here has been used to produce hundreds of independent, transgenic plant lines and we show that a large proportion of these lines contain single copies of the *luc *gene.

**Conclusion:**

This protocol demonstrates significant improvements in both efficiency and ease of use over existing barley transformation methods. This opens up opportunities for the development of functional genomics resources in barley.

## Background

The genetic transformation of cereal crops has been widely studied as a tool for crop improvement and as a vital part of the development of functional genomics resources. Barley ranks fourth among the cereals in quantity produced and is grown in around 100 countries worldwide. As well as being an important crop in its own right, barley has also been suggested as a valuable diploid model for the more complex, hexaploid cereal crop, wheat. For either purpose, a highly efficient barley transformation system is required. The first fertile transgenic barley plants were produced by particle bombardment of immature embryos [1]. This was followed by reports of *Agrobacterium*-mediated transformation of barley immature embryos [2]. Alternative target tissues have also been used for barley transformation including microspores [3] and shoot meristematic cultures [4]. However, immature embryos still remain the target tissue of choice in barley. In a comparison of particle bombardment and *Agrobacterium*-mediated techniques, it was shown that *Agrobacterium*-mediated transformation was more efficient and led to the production of transgenic lines with lower numbers of transgene copies. In addition, lines produced using *Agrobacterium*-mediated techniques showed more stable transgene expression over generations and fewer cases of transgene silencing [5].

The development of functional genomics resources in rice is far in advance of those in wheat and barley due to the fact that very high-throughput transformation systems are available for rice [6]. This means that resources such as large T-DNA insertion populations can be developed [7]. In some recent reports of *Agrobacterium*-mediated barley transformation, efficiencies of 5.4% were achieved by Lange *et al*. [8], while Shrawat *et al. *[9] obtained frequencies ranging from 2.6 up to 6.7%. Matthews *et al*., [10] described barley transformation frequencies in the range 2–12% and Murray *et al.*, [11] obtained average efficiencies between 4.4 and 9.2%. A more recent report describes a transformation efficiency of 21.7%, averaged across two experiments starting with a total of 600 immature embryos [12]. However, a number of different modifications were employed during the study, making the method rather complex. Without reproducible, simple and highly efficient transformation technologies for barley, the development of resources, such as T-DNA insertion mutants, requiring thousands of independent transgenic lines, would extremely difficult. For a recent review of *Agrobacterium*-mediated transformation of cereals detailing the efficiencies for the different crops see [13].

A major constraint in the improvement of barley transformation efficiencies is the ability to successfully regenerate plants from transformed callus. It has previously been shown that plant transformation efficiencies have the potential to be as high as 40% if all transgenic callus lines, produced by particle bombardment, regenerated into independently transformed plants [14]. There have been a great number of studies examining factors affecting cereal transformation efficiency. Most commonly these have examined factors such as medium composition, *Agrobacterium *inoculation procedure, *Agrobacterium *strain and vector, selective agent, explant type and size. In barley, the effect of including an intermediate culture step before regeneration was examined [15]. The seasonal effect on tissue culture and plant regeneration in barley was examined by Sharma *et al*. [16]. Shrawat *et al*. [9] examined a range of factors including pre-culture of immature embryos, co-cultivation period and temperature, the presence of acetosyringone, pH of the medium, sonication and vacuum infiltration to assist *Agrobacterium *inoculation and the timing of the introduction of selection. Hensel *et al*. [12] examined embryo treatments, addition of acetosyringone and L-cysteine and *Agrobacterium *strain. A number of studies have described the beneficial effects of including increased levels of copper in culture medium, compared to the standard medium of Murashige and Skoog [17]. Improved plant regeneration in barley by using increased levels of copper was described by Dahleen [18]. High levels of copper have also been reported to have a beneficial effect in other species [19].

In the present study we examined the timing of copper addition in order to significantly improve regeneration rates. This regeneration protocol was then combined with a very simple transformation procedure that did not need any special embryo treatments, to develop a robust, straightforward and highly efficient method. The improved protocol has yielded large numbers of independently transformed barley plants with average transformation efficiencies of 25%, paving the way for the development of functional genomics resources in barley.

## Methods

### Plant material and immature embryo isolation

Donor plants of the spring barley, Golden Promise, were grown under controlled environment conditions with 15°C day and 12°C night temperatures as previously described [20]. Humidity was 80% and light levels 500 μmol.m^-2^.s^-1 ^at the mature plant canopy level provided by metal halide lamps (HQI) supplemented with tungsten bulbs. Immature barley spikes were collected when the immature embryos were 1.5–2 mm in diameter. Immature seeds were removed from the spikes and sterilised as previously described [20]. The immature embryos were exposed using fine forceps and the embryonic axis removed. The embryos were then plated scutellum side up on callus induction (CI) medium containing 4.3 g l^-1 ^Murashige & Skoog plant salt base (Duchefa), 30 g l^-1 ^Maltose, 1.0 g l^-1 ^Casein hydrolysate, 350 mg l^-1 ^Myo-inositol, 690 mg l^-1 ^Proline, 1.0 mg l^-1 ^Thiamine HCl, 2.5 mg l^-1 ^Dicamba (Sigma-Aldrich) and 3.5 g l^-1 ^Phytagel, with 25 embryos in each 9 cm Petri dish. 1.25 mg l^-1 ^CuSO_4_.5H_2_O was added where experiments included additional copper in the callus induction phase. Additional copper was present in the CI media of all six transformation experiments described. All media components used throughout the tissue culture process, with the exception of phytagel, were made as a double-concentrate and filter-sterilised. Phytagel was made up as a double concentrate (in H_2_O) and autoclaved. After autoclaving in H_2_O the phytagel could be stored at room temperature until required, and then heated to 60°C prior to mixing with the filter-sterilised media components.

### Plasmids and bacterial strain

**Figure 1:**
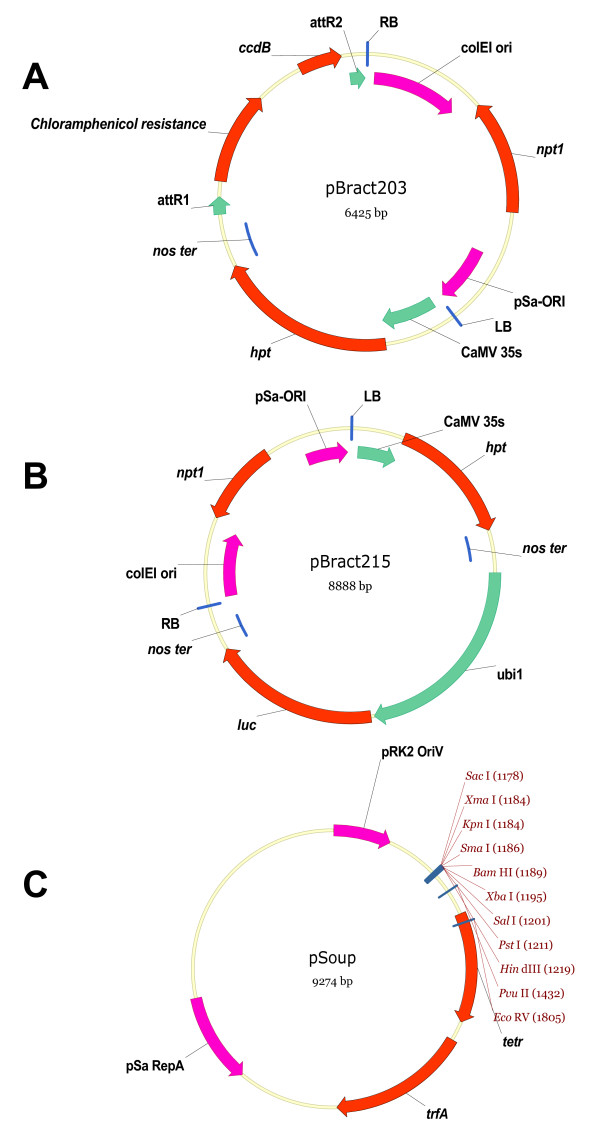
**Vectors used.** (a) and (b): pBract vectors. (a). pBract203 destination vector containing the *hpt *gene under the control of a CaMV 35s promoter together with the chlormaphenicol resistance gene and the negative selection *ccdB *gene flanked by attR Gateway recombination sites. (b). pBract215 expression vector, created by cloning a luciferase cassette into pBract203 (via Gateway recombination). This construct contains the *hpt *gene under the control of a CaMV 35s promoter together with the luciferase gene under the control of a maize ubiquitin promoter (ubi1). (c): pSoup containing pSa-*RepA *(a component of the *Agrobacterium *replication origin), the replicase gene *trfA*, a tetracycline resistance gene (*tetR*), a broad-host range origin of replication (pRK2) and a multiple cloning site.

*Agrobacterium *strain AGL1 containing one of three pBract vectors (pBract215, pBract216 or pBract217) was used in all experiments. The vectors pBract215, pBract216 and pBract217 all derive from the Gateway destination vector pBract203, which features a T-strand region containing the *hpt *gene (conferring hygromycin resistance) under the control of a CaMV 35s promoter and a negative selection gene (*ccdB*) flanked by Gateway recombination sites to assist the cloning of genes of interest (Figure 1). In pBract215, pBract216 and pBract217, the *ccdB *gene has been replaced by the firefly luciferase gene (*luc*) driven by the maize Ubi1 promoter. These vectors are all the same apart from the intron composition of their luciferase genes. pBract216 and pBract217 contain the maize *RpoT *intron 4 and *Arabidopsis UBQ10 *intron 1 respectively within the coding sequence of the luciferase gene at position +165. The pBract vectors are detailed on the bract website [21]. All pBract vectors are based on pGreen [22] and therefore need to be co-transformed into *Agrobacterium *with the helper plasmid pSoup. To enable the small size of pGreen, the pSa origin of replication required for replication in *Agrobacterium*, is separated into its' two distinct functions. The replication origin (ori) is present on pGreen, and the trans-acting replicase gene (*RepA*) is present on pSoup. Both vectors are required in *Agrobacterium *for pGreen to replicate. Maps of pBract203, pBract215 and pSoup are shown in Figure 1.

### Agrobacterium-mediated transformation

The pBract vectors were independently electroporated into *Agrobacterium *AGL1 cells along with pSoup. Each electroporation was set up by adding 1 μl of pBract construct DNA (100 ng/μl) and 1 μl of pSoup DNA (100 ng/μl) to 40 μl of electrocompetent AGL1 cells on ice. The cells and DNA were gently mixed, and then transferred to a pre-chilled cuvette (with 2 mm separation). Electroporation was carried out using a Biorad GenePulser, with the settings: 2.50 kv, 25 μFD, 400 Ohms. The time constant was between 8.9 and 9.2 ms. The cells were recovered by adding 100 μl LB media (10 g l^-1 ^tryptone, 5 g l^-1 ^yeast extract and 10 g l^-1 ^NaCl) and then transferring the contents of the cuvette to 2 ml of LB. This was gently shaken at room temperature (approximately 24°C) for 2 hours. One hundred microlitres of the electroporation was plated on LB solidified with 1% agar and supplemented with 50 μg ml^-1 ^kanamycin and 50 μg ml^-1^rifampicin. These plates were incubated at 28°C for 48 hours.

The method of Tingay *et al*. [2] was used to prepare a standard *Agrobacterium *inoculum for transformation. A 400 μl aliquot of standard inoculum was removed from -80°C storage, added to 10 ml of MG/L [23] medium without antibiotics and incubated on a shaker at 180 rpm at 28°C overnight. This full strength culture was used to inoculate the prepared immature embryos. A small drop of *Agrobacterium *suspension was added to each of the immature embryos on a plate. The plate was then tilted to allow any excess *Agrobacterium *suspension to run off. Immature embryos were then gently dragged across the surface of the medium (to remove excess *Agrobacterium*) before being transferred to a fresh CI plate, scutellum side down. Embryos were co-cultivated for 3 days at 23–24°C in the dark.

### Selection of transformed plants

After co-cultivation, embryos were transferred to fresh CI plates containing 50 mg l^-1 ^hygromycin and 160 mg l^-1 ^Timentin (Duchefa). Our standard *Agrobacterium*-mediated transformation protocol [5] involved culture for four to six weeks on CI medium followed by two weeks on a transition medium with increased levels of copper similar to that described by Cho *et al*. [15], before transfer to regeneration medium. The main difference between our protocol and that of Cho *et al*. [15] was that our transition medium was based on the regeneration medium, not on the callus induction medium. In the improved protocol described here, copper at 1.25 mg l^-1 ^was added to all CI medium following the co-cultivation step. Embryos were sub-cultured onto fresh CI selection plates every 2 weeks and kept in the dark at 24°C. After 4–6 weeks, embryos were transferred to transition medium containing 2.7 g l^-1 ^Murashige & Skoog modified plant salt base (without NH_4_NO_3_) (Duchefa), 20 g l^-1 ^Maltose, 165 mg l^-1 ^NH_4_NO_3_, 750 mg l^-1 ^Glutamine, 100 mg l^-1 ^Myo-inositol, 0.4 mg l^-1 ^Thiamine HCl, 1.25 mg l^-1 ^CuSO_4_.5H_2_O, 2.5 mg l^-1 ^2, 4-Dichlorophenoxy acetic acid (2,4-D) (Duchefa), 0.1 mg l^-1 ^6-Benzylaminopurine (BAP), 3.5 g l^-1 ^Phytagel, 50 mg l^-1^Hygromycin and 160 mg l^-1 ^Timentin in low light. Low light levels (75 μmol.m^-2^.s^-1^) were achieved by placing the plates under light conditions in a tissue culture room but covering the plates with a thin sheet of paper. After a further 2 weeks, embryo derived callus was transferred to regeneration medium in full light (140 μmol.m^-2^.s^-1^) at 24°C, keeping all callus from a single embryo together. Regeneration medium was the same as the transition medium but without additional copper, 2,4-D or BAP. Once regenerated plants had shoots of 2–3 cm in length they were transferred to glass culture tubes containing CI medium, without dicamba or any other growth regulators but still containing 50 mg l^-1 ^hygromycin and 160 mg l^-1 ^Timentin. Transformed plants developed a strong root system in the hygromycin containing medium in 1–2 weeks and were then transferred to soil and grown under the same conditions as the donor plants.

### Luciferase assays

Luciferase assays were performed using 2 cm leaf samples taken from regenerated plantlets growing in culture tubes containing CI medium with selection. Luciferase activity was quantified according to the method of Harwood *et al*. [14].

### PCR analysis

PCR analysis was carried out to confirm the presence of the *hpt *gene for lines deriving from transformation with the constructs pBract216, pBract215 and pBract217 which were shown to have no luciferase expression. DNA was extracted (by the John Innes Genome Laboratory) from 50 mg of leaf tissue using the Qiagen DNeAsy 96 Plant kit (Q69181) system following the manufacturer's instructions. A 917-bp fragment of the *hpt *gene was amplified using the *aphIV *primers described by Vain *et al. *[24]. Reactions were composed of 17 μl ABgene 1.1× ReddyMix™ PCR Master Mix (1.5 mM MgCl_2_), 0.5 μM of each primer and 20 – 35 ng template DNA. PCR was carried out using a Peltier Thermal cycler PTC-200 (MJ Research), with the conditions 95°C for 1 minute, 95°C for 30 seconds, 60°C for 30 seconds, 72°C for 1 minute, return to step 2 30 times, 72°C for 10 minutes. PCR products were separated on 1% agarose gels (containing 10 μg of ethidium bromide per 100 ml) using gel electrophoresis.

### Statistical analyses

Differences in transformation frequencies were assessed using chi-square analysis. Two-way tables giving the numbers of transformed/untransformed embryos for each variable were entered into Genstat (version 9.1.0.147). For each 2-way table, Genstat computed a Pearson chi-square value, and from this generated the *p*-value. Significance was assessed at the 5% level.

### Quantitative real-time PCR for determining transgene copy number

DNA was extracted by the same method used for the *hpt *PCR (described above). Quantitative real-time PCR was carried out by iDna Genetics using luciferase and *CO2 *(Constans-like, AF490469) gene specific primers and probes. Primers were designed to the target sequences using Applied Biosystems software Primer Express, with the TaqMan Probe and Primer design module (Table 1). The reactions used ABGene Absolute QPCR Rox Mix (Cat number AB1139). The luciferase gene and the *CO2 *gene were assayed in multiplex. The probes and primer final concentrations were 200 nM. The assay contained 5 μl of DNA solution, and was optimised for final DNA concentrations 1.25 to 10 ng/μl (6.25 to 50 ng DNA in the assay). PCRs were carried out in an Applied Biosystems ABI7900 equipped with a 384 place plate. The detectors used were FAM-TAMRA and VIC-TAMRA with Rox internal passive reference, and no 9600 emulation. The PCR cycling conditions were 50°C 2 minutes hold, 95°C 10 minutes (enzyme activation), 40 cycles of 95°C 15 seconds, 60°C 60 seconds.

**Table 1:** Primer and probe sequences used for quantitative real-time PCR for determining copy number of the luciferase gene.

	Luciferase		Barley *CONSTANS*-like *CO2 *gene^a^	
Primers	LucF1	CCGCCTGAAGTCTCTGATTAAGTAC	HvCon2F1	TGCTAACCGTGTGGCATCAC
	LucR1	GACACCTGCGTCGAAGATGTT	HvCon2R1	GGTACATAGTGCTGCTGCATCTG
Probes	LucP	6FAM-AGGCTATCAGGTGGCTCCCGCTG-TAMRA	HvCon2P	VIC-CATGAGCGTGTGCGTGTCTGCG-TAMRA

^a^The barley *CONSTANS*-like *CO2 *gene was used as an internal positive control.

### Southern analysis

Eight lines transformed with pBract216 were subject to Southern analysis. For each line, fifteen micrograms of genomic DNA were digested with 40 units of BamHI (which cuts once within *hpt *and once between ubi1 and *luc*) and separated on a 0.8% agarose gel. An alkaline Southern blot was assembled using a Hybond-N+ membrane (Amersham) according to manufacturer's instructions. DNA was fixed to the membrane by cross-linking in a UV Stratalinker 2400 (Stratagene). Probe DNA was produced by PCR using the primers LUCF (5'-GCCGGTGTTGGGCGCGTT-3') and LUCR (5'-GCGGGAAGTTCACCGGCG-3') to amplify a 1176 bp fragment of the *luc *coding sequence. The probe was labelled with ^32^P α-dCTP prior to hybridisation. The blot was imaged using a Typhoon 9200 Phosphorimager (Amersham).

## Results

### 1. Development of the improved Agrobacterium-mediated barley transformation protocol

Development of the high-throughput *Agrobacterium*-mediated barley transformation system was dependent on the availability of immature embryos from good quality plants grown under controlled conditions. All immature embryos used in the present study were from plants of the spring genotype Golden Promise, grown under controlled conditions. Plants were not sprayed with fungicides or insecticides at any stage of growth. Our earlier work indicated that poor transformation results could often be linked to poor quality donor material or to occasions when plants had been sprayed (unpublished). In addition, particular care was taken with the watering regime so that the plants were never too dry or overwatered. Starting with good quality immature embryos, an experiment was initiated to look at the effect of additional copper in the culture medium on regeneration efficiency.

**Figure 2:**
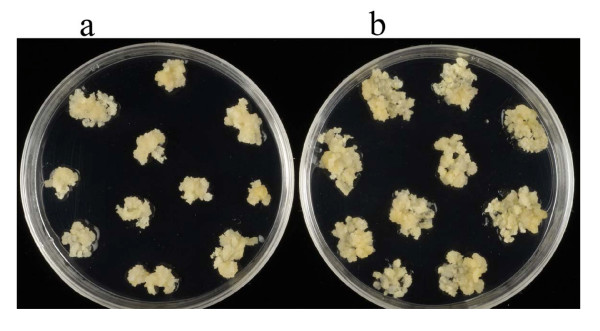
**The effect of additional copper on the growth of callus from immature barley embryos.** (a) and (b) show immature embryo derived callus after 27 days culture on callus induction medium. (a): standard callus induction medium. (b): Callus induction medium with 5 μM CuSO_4_.

Our standard protocol was compared to a method that included additional copper (5 μM CuSO_4_) in the CI medium as well as in the transition medium. This study was carried out without any *Agrobacterium *treatment to examine regeneration ability from immature embryos only. Fifty-eight immature embryos with the embryonic axis removed were used in this study and were split between two different culture regimes. Thirty embryos were cultured on callus induction medium (CI) (standard protocol) and twenty-eight embryos were cultured on callus induction medium supplemented with 5 μM CuSO_4 _(CI + Cu). The appearance of the embryo derived callus after 27 days of culture is shown in Figure 2. There was more callus growth on the CI medium with copper (Figure 2b) than on the standard CI medium (Figure 2a). In addition, detailed examination of the callus revealed that as well as showing faster growth, the callus on the copper containing medium appeared more embryogenic. After four weeks on callus induction medium, all callus lines were transferred to transition medium for two weeks and then to regeneration medium. All callus derived from each embryo was kept separate and after 4 weeks on regeneration medium the shoots derived from each embryo were removed and counted.

**Figure 3:**
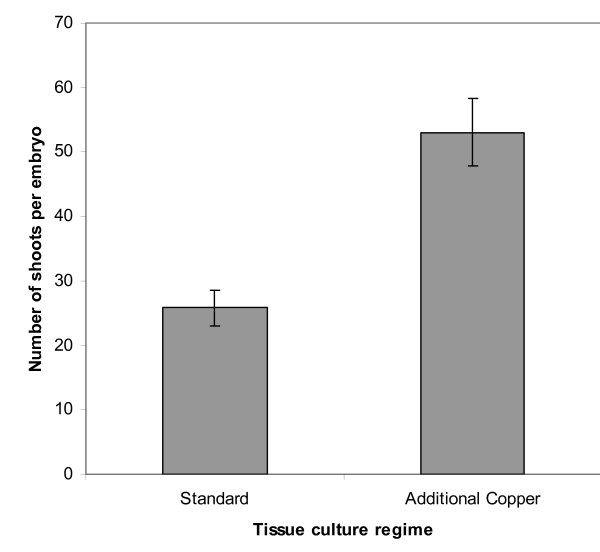
**The effect of copper treatments on the number of shoots regenerated per embryo without transformation.** Each bar represents the average number of shoots per embryo (recorded after 10 weeks in tissue culture) from 20–30 individual embryos. 'Standard' tissue culture regime: weeks 1–4 callus induction (CI) medium with no additional copper. 'Additional copper' tissue culture regime: weeks 1–4 CI + 5 μM CuSO_4_. Callus from both treatments was transferred to standard transition media (which includes CuSO_4_) after 4 weeks and standard regeneration medium after a further 2 weeks.

Figure 3 shows the average number of shoots regenerated per embryo with the two different tissue culture regimes. The standard protocol, gave an average of 26 shoots per embryo. The improved protocol, containing copper in the CI medium, gave an average of 53 shoots per embryo. Adding the additional copper during callus induction therefore led to approximately double the number of regenerated shoots per embryo up to the point when the shoots were scored. The shoots derived from embryos cultured on CI + Cu were also larger than those cultured without copper. It appears that the regeneration process was accelerated by the presence of copper in the CI medium as embryogenic callus formed earlier leading to the more rapid regeneration of plants.

These two protocols were compared in some small preliminary transformation experiments (data not shown). The number of transformed callus lines obtained was unaffected by the additional copper, however the number of transformed plants obtained as a proportion of the number of starting embryos was significantly higher when copper was added to the callus induction medium. Improved plant regeneration therefore appeared to increase transformation efficiency. This initial finding was further examined in large scale experiments described below.

### 2. Performance of the improved protocol and investigation into the timing of embryo inoculation

Six transformation experiments (A – F, detailed in Table 2) were carried out to test the improved barley transformation protocol, using a total of 1750 immature embryos. These experiments utilised the three constructs, pBract215, 216 and 217, which are identical apart from the intron content of their luciferase gene. For these experiments, the improved regeneration regime (with 5 μM CuSO_4 _in CI media) was used. In addition, all culture media were filter sterilised and embryos were collected from healthy, well maintained plants that had not been sprayed at any stage of growth. These were the key differences from our previous protocol [5]. Some previous studies have recommended using freshly isolated embryos whereas others have included pre-culture of embryos prior to inoculation. In the present experiments, the effect of modifying the timing of embryo isolation/inoculation upon transformation efficiency was investigated by inoculating embryos on both the same day as isolation and the day after isolation in four of the six experiments.

**Table 2:** Detailed results from six independent transformation experiments using the improved protocol.

Experiment	Construct	Embryo inoculation time	Number of embryos	Independent hygromycin-resistant plants	Transformation Efficiency^a^
A	pBract216	DA	75	20	26.7%
	pBract215	DA	50	15	30.0%
		SD	25	5	20.0%
	pBract217	DA	50	16	32.0%
		SD	25	5	20.0%

B	pBract216	DA	25	1	4.0%
		SD	75	23	30.7%
	pBract215	DA	25	8	32.0%
		SD	75	22	29.3%
	pBract217	DA	25	7	28.0%
		SD	75	16	21.3%

C	pBract216	DA	50	9	18.0%
		SD	75	20	26.7%
	pBract215	DA	75	17	22.7%
		SD	50	9	18.0%
	pBract217	DA	75	24	32.0%
		SD	50	16	32.0%

D	pBract216	SD	75	12	16.0%
	pBract215	SD	75	22	29.3%
	pBract217	SD	75	10	13.3%

E	pBract216	SD	100	17	17.0%
	pBract215	SD	75	19	25.3%
	pBract217	SD	75	14	18.7%

F	pBract216	DA	25	2	8.0%
		SD	100	25	25.0%
	pBract215	DA	50	18	36.0%
		SD	75	22	29.3%
	pBract217	DA	50	13	26.0%
		SD	75	25	33.3%

Total			1750	432	24.7%

DA = day after (embryos inoculated the day after isolation); SD = same day (embryos isolated and inoculated on the same day). ^a^Transformation efficiency is given as the number of independent transformed lines yielding green, hygromycin resistant plants as a percentage of the number of immature embryos treated.

### 2.1 Timing of embryo inoculation

Experiments A, B, C and F (Table 2) were each performed using embryos inoculated on both the same day and the day after isolation. These experiments showed no significant effect of embryo isolation/inoculation timing on transformation efficiency (*p *= 0.342) (Table 3). Combining the results of all six experiments, 150 independent transformed lines were created from 575 embryos inoculated on the day after isolation (giving a transformation efficiency of 26.1%) and 282 independent transformed lines were produced from 1175 embryos inoculated on the same day as isolation (giving a transformation efficiency of 24.0%).

**Table 3:** Summary of the average transformation efficiencies obtained for the six experiments, three constructs and two timings of *Agrobacterium *inoculation using the improved protocol.

		Average transformation efficiency
Experiments:	A	27.1%
	B	25.7%
	C	25.3%
	D	19.6%
	E	20.0%
	F	28.0%

Constructs:	pBract216	21.5%
	pBract215	27.3%
	pBract217	25.4%

Timing of inoculation:	DA	26.1%
	SD	24.0%

DA = day after (embryos inoculated the day after isolation); SD = same day (embryos isolated and inoculated on the same day).

### 2.2 Transformation efficiency of improved protocol

The improved method was applied to a total of 1750 immature embryos, and of these, 432 were successfully transformed into independent hygromycin-resistant plants, giving an average transformation efficiency of 24.7% (Table 2). Plants producing strong roots in hygromycin containing medium were found to be transformed in 98.5% of cases. There was no significant difference in transformation efficiency between constructs (*p *= 0.062) and between experiments (*p *= 0.098) (Table 3). The transformed status of 333 independent lines was checked by analysis of luciferase expression (data not shown). Of these lines, 95.5% were expressing the luciferase gene. Two thirds of the 4.5% of lines not expressing luciferase were subsequently shown (by PCR) to contain the hygromycin resistance gene (*hpt*) – validating their transformed status. The remaining lines (1.5% of the total analysed for luciferase expression) did not contain the *hpt *gene and were therefore considered untransformed. These 5 lines were omitted from the transformation efficiency calculations (Tables 2 and 3) and their possible origin is discussed later. Plants from 328 of the independent lines were grown to maturity and of these 321 produced seed giving a 98% fertility rate.

### 2.3 Transgene copy number of transformed lines produced

**Figure 4:**
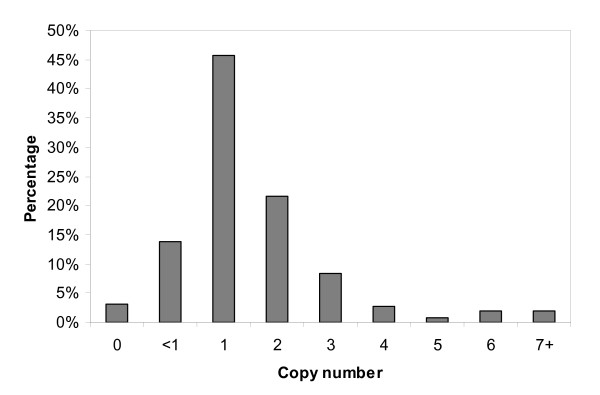
**Copy numbers of the luciferase gene within transformed plants, determined by quantitative real-time PCR, given as a percentage of the 260 lines analysed.** Note that '<1' refers to samples which gave a positive quantitative real-time PCR signal for luciferase and the internal control gene, but the luciferase signal occurred later than for single copy lines (a later signal normally implies less starting template) indicating there may be a problem with gene rearrangements.

**Figure 5:**
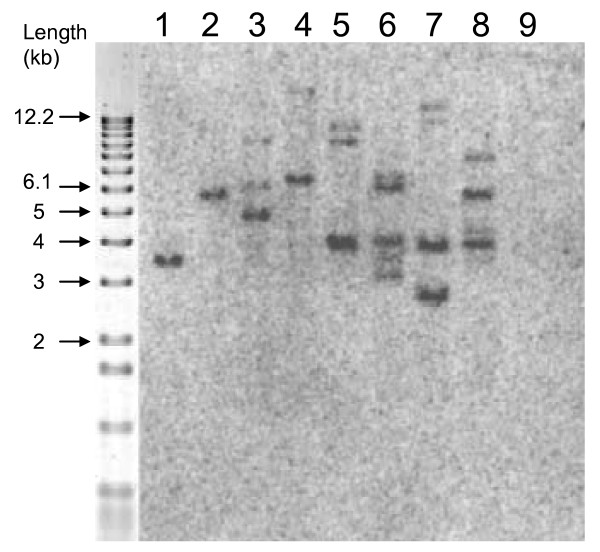
**Southern blot analysis of eight independently-transformed pBract216 lines.** Genomic DNA was digested with BamHI and probed with a 1176 bp fragment of luciferase DNA. The lanes 1–8 correspond to lines 90-04-01, 90-06-01, 70-05-01, 70-07-01, 70-21-01, 84-07-01, 87-07-01, and 81-14-01 respectively. Lane 9 corresponds to a wild-type Golden Promise control.

Luciferase copy number was determined for 260 independent hygromycin-resistant lines using real-time PCR. The distribution of copy numbers obtained is given in Figure 4. Of the transformed plant lines analysed, 8 (3%) did not contain an intact copy of the luciferase gene. These lines were not escapes, as they were shown by PCR to contain the *hpt *gene. Forty-six percent of the transformed lines contained a single copy of the luciferase gene, and only 8% of the lines contained more than three copies. The distribution of copy numbers obtained was consistent across the three constructs used. Southern analysis was performed to check the *luc *copy number and integration pattern in eight T_0 _lines transformed with pBract216 (Figure 5). For seven of the lines, the number of T-DNA insertion sites identified by Southern analysis was less than or equal to the number of *luc *copies predicted by qPCR, as would be expected (Table 4).

**Table 4:** A comparison of qPCR and Southern analysis results.

Plant line	qPCR copy number estimation	Number of bands obtained in Southern analysis
90-04-01	1	1
90-06-01	1	1
70-05-01	2	3
70-07-01	2	2
70-21-01	4	3
84-07-01	5	4
87-07-01	6	4
81-14-01	7	4

## Discussion

Transformation efficiency has for many years been a limiting factor in studies requiring the production of large numbers of transgenic barley plants. Using biolistic-based methods to produce transgenic barley, it was demonstrated that the main block to achieving high transformation efficiencies was the ability to regenerate plants from transformed callus lines [14]. Following the first reports of *Agrobacterium*-mediated barley transformation [2], a comparison of the two methods revealed that the *Agrobacterium*-mediated method offered advantages in terms of lower copy number and more stable transgene expression [5]. In the current study we have improved the *Agrobacterium*-mediated barley transformation system, in particular the regeneration of green plants from transformed callus, to give a highly efficient, straightforward and reproducible method that yields average transformation efficiencies of 25%. Recent experiments in our laboratory have reached transformation efficiencies of over 50% indicating that average efficiencies can be increased further.

In the development of the high-throughput transformation method, an improvement was made by adjusting the timing of the addition of copper to the culture medium. The positive effect of including the correct concentration of copper on plant regeneration has been well documented. Purnhauser and Gyulai [25] demonstrated enhanced shoot regeneration from calli of wheat and triticale following the inclusion of copper at a higher concentration than that originally used in the medium of Murashige and Skoog [17]. The positive effect of higher levels of copper on regeneration from barley callus cultures was described by Dahleen [18] and both Bregitzer *et al*. [26] and Nuutila *et al*. [27] showed that the addition of 5 μM copper in tissue culture media gave the highest green plant production in a range of barley cultivars. Other crops have also shown improved regeneration in response to higher levels of copper for example rice [28] and sorghum [29]. The use of increased levels of copper to aid the recovery of transgenic barley plants was described by Cho *et al *[15]. In this study the authors described the inclusion of an intermediate culture step, which included increased levels of copper, before the plant regeneration stage. The use of the intermediate culture step was adopted in our previous work with the *Agrobacterium*-mediated barley transformation system [5], however, our intermediate medium was based on regeneration medium not on the callus induction medium as used by Cho *et al*. [15]. In developing the high-throughput method described in the current study, it was found that inclusion of 5 μM copper in all callus induction stages and during the intermediate step resulted in twice the number of regenerated shoots per embryo compared to a culture regime where the additional copper was only added during the intermediate step.

In this study, all media were filter-sterilised in order to prevent adverse effects associated with autoclaving. Bregitzer *et al*. [26] have previously shown better green plant regeneration in barley when the carbon source and iron source in the culture medium were autoclaved separately from other media components. In addition, the degradation of sugars during autoclaving is known to have adverse effects on plant cultures, for example [30]. It is likely that using filter-sterilised media gave improved results due to the avoidance of unwanted interactions between media components and the production of toxic products from the breakdown of sugars.

In the study described here, no significant difference in transformation efficiency was seen between embryos inoculated on the same day as isolation compared to those inoculated the day after isolation. This contributes flexibility to the methodology, enabling isolation and inoculation to be spread over two days, or carried out on the same day. Some studies recommended the use of acetosyringone during co-cultivation [9,12] however we have not found this necessary to achieve high transformation efficiencies. One key requirement for high transformation efficiencies is good quality plants grown under controlled conditions from which to collect immature embryos. In the past we have noticed that poor transformation results appear to be linked to poor quality donor plants or to occasions when the plants were sprayed with fungicide or insecticide. In the improved method we have not applied any spray treatments to the plants. We have also paid particular attention to care of the donor plants and the plant watering regime. Another key feature of the method was the use of constructs from the pBract series [21]. These constructs have proved to be extremely efficient but no direct comparison has been made to other available constructs.

Using the high-throughput transformation method, 432 independent transformed lines, yielding green plants, were recovered from 1750 treated immature embryos giving an average transformation efficiency of 24.7%. The efficiency of the method means that one small experiment involving 100 embryos is sufficient to generate over 20 independent lines. This makes the barley transformation method a very attractive research tool for over-expression or silencing of genes of interest in a cereal crop plant. The lack of variation in transformation efficiency between independent experiments and the lack of failed experiments also adds to the utility of the method as reliable research tool. Advantages of the method also include the very simple transformation and tissue culture procedures with no additional treatments such as vacuum infiltration [12] or sonication [9] being necessary. Also there is no time consuming visual selection of callus and the callus from each embryo does not need to be split up during culture making sub-cultures very quick and straightforward.

Once transferred to transition medium, the transformed lines are obvious as rapidly growing lines that develop green sectors within a few days of transfer to the hygromycin containing transition medium. Escapes using hygromycin selection are very rare as plants that regenerate and form roots in hygromycin containing medium are almost always transformed. The five plants out of 333 that were found not to contain the hygromycin gene in the present study were likely to have been derived from a non-transformed shoot growing very close to, and maintained by, a transformed shoot so that both shoots were transferred to a tube for rooting together. During transfer to soil, multiple shoots were separated and only one plant from each line was transferred to soil. Thus it is possible that under these circumstances a non-transformed plant was chosen. From the 432 independent lines, plants from 328 lines were grown to maturity and 321 of these produced seed meaning that 98% of the lines were fertile. The same 328 lines were assayed for luciferase expression and 95.5% of these hygromycin resistant lines also expressed the luciferase gene present on the same T-DNA thus giving a very high co-expression rate. Fifteen single copy T_0 _lines were taken through to the T_2 _generation and these displayed stable luciferase expression (data not shown).

Analysis of luciferase gene copy number in 260 independently transformed lines revealed that 46% of the lines contained a single copy of the luciferase gene and 22% contained two copies of the transgene. In a study of more than 200 transgenic rice lines, 30–40% were found to have a single copy of the transgene [6], slightly lower than found in the current study. Only 8% of lines in our study contained more than three copies of the luciferase gene. Fourteen per cent of the 260 samples assessed for transgene copy number gave a positive quantitative real-time PCR signal for luciferase and the internal control gene, but the luciferase signal occurred later than for single copy lines. This could be explained by gene re-arrangements leading to less efficient amplification in these lines. Eight transgenic lines were subjected to Southern analysis. Seven of these produced a number of Southern bands that was equal to or less than the number of *luc *copies predicted by qPCR. An equal number would be expected if each of the transgenes integrated separately at independent insertion sites. A lower number of insertion sites relative to copy-number would be expected if tandem or inverted T-DNA repeats were present within a single insertion site. Tandem T-DNA insertions have previously been identified in approximately 50% of barley transformants [31]. One line was found to have three insertion sites, but was predicted by qPCR to contain only two copies of *luc*. It is possible that within this line, one copy of *luc *featured mutations within one or both of the qPCR primer binding-sites. In general, there was a good agreement between the Southern analysis and qPCR, in particular for the single copy lines.

## Conclusion

High-throughput barley transformation leads to the possibility of the development of improved functional genomics tools for use in both wheat and barley crop improvement. Over-expression or silencing of target genes is now extremely straightforward in barley. The close relationship between wheat and barley means that studies in barley can provide valuable information for applications in wheat where *Agrobacterium*-mediated transformation is far more difficult. In addition, with routine 25% transformation efficiencies, the development of tools such as T-DNA insertion populations in barley is a possibility. Using the improved method, one person could make at least 2,500 T-DNA insertion lines per year. It is likely that with further optimisation, even higher efficiencies will be achieved opening up even more possibilities for transformation based resources in barley.

## Competing interests

The authors declare that they have no competing interests.

## Authors' contributions

JB carried out the six transformation experiments, performed the luciferase assays, and carried out the *hpt *PCRs. SA carried out the regeneration study and some other preliminary experiments which contributed to the improved transformation method. MS performed a preliminary study to examine the effect of additional copper in the culture media. JS assisted with statistical analysis of the data. WH conceived the study, participated in its design and coordination and was involved in drafting the manuscript. All authors have read and approved the final manuscript.
